# Supplementary material for: GAL3ST1 Deficiency Reduces Epithelial–Mesenchymal Transition and Tumorigenic Capacity in a Cholangiocarcinoma Cell Line
Source: Int J Mol Sci. 2024 Jul 2;25(13):7279. doi: 10.3390/ijms25137279 (PMC11242791; doi:10.3390/ijms25137279)
Supplement: Supplementary file 1 [file ijms-25-07279-s001.zip › Supplementary material_v2-proofread.pdf]

Table S1. List of primers used for qRT-PCR

Gene	Forward primers (5'-3')	Reverse primers (5'-3')
36B4	CCTCGTGGAAGTGACATCGT	ATCTGCTTGGAGCCCACATT
GAL3ST1	AACCACATGCGCTTCCACTA	GTCTTGCAGGAACTCGGTCA
HK2	AGACGAGAGTTTCCTGGTCTC	GATGGCCTTCCGGATCAGAG
PGK1	AGCGGGTCGTTATGAGAGTC	TGGGACAGCAGCCTTAATCC
HIF1A	TCACCTGAGCCTAATAGTCC	AAATGGGTTCTTTGCTTCTG
ZO-1	AGGGGCAGTGGTGGTTTTCTGTTCTTTC	GCAGAGGTCAAAGTTCAAGGCTCAAGAGG
OCLN	TCAGGGAATATCCACCTATCACTTCAG	CATCAGCAGCAGCCATGTACTCTTCAC
E-cadherin	CACCTGGAGAGAGGCCGCGT	AACGGAGGCCTGATGGGGCG
CLDN-1	GGGCTGCAGCTGTTGGGCTT	GGG TTG CTT GCA ATG TGC TGC T
Fibronectin	CAGTGGGAGACCTCGAGAAG	GTCCCTCGGAACATCAGAAA
Vimentin	GAGAACTTTGCCGTTGAAGC	TCCAGCAGCTTCCTGTAGGT

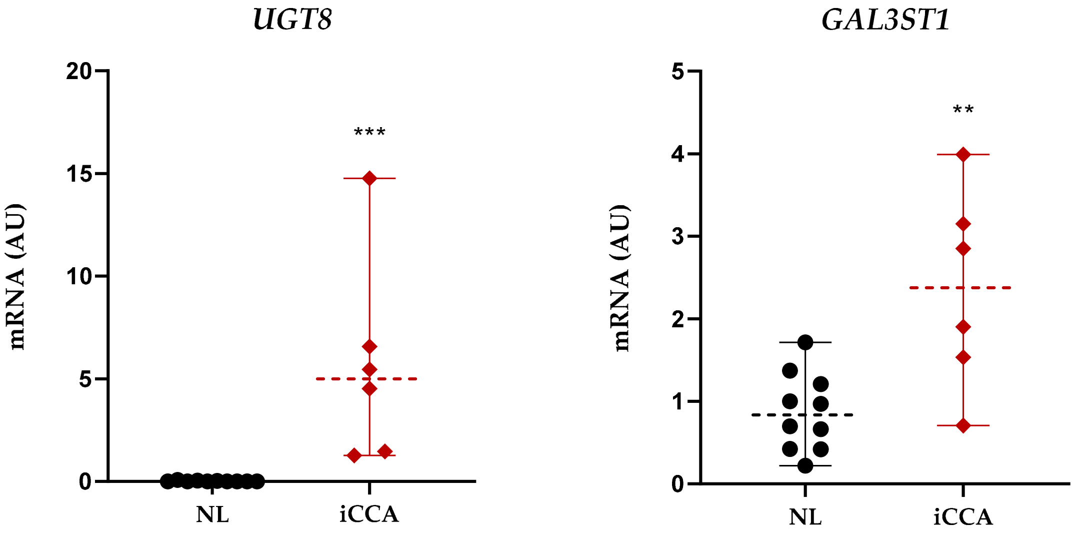

Figure S1A. UGT8 and GAL3ST1 expression in human iCCA tumor specimens. Relative mRNA levels of *UGT8* and *GAL3ST1* in intrahepatic CCA (iCCA) tissue (n = 6) compared to non-malignant liver (NL) tissue (n = 10). Data are shown as median with range $^{**}p < 0.01$, $^{***}p < 0.001$.

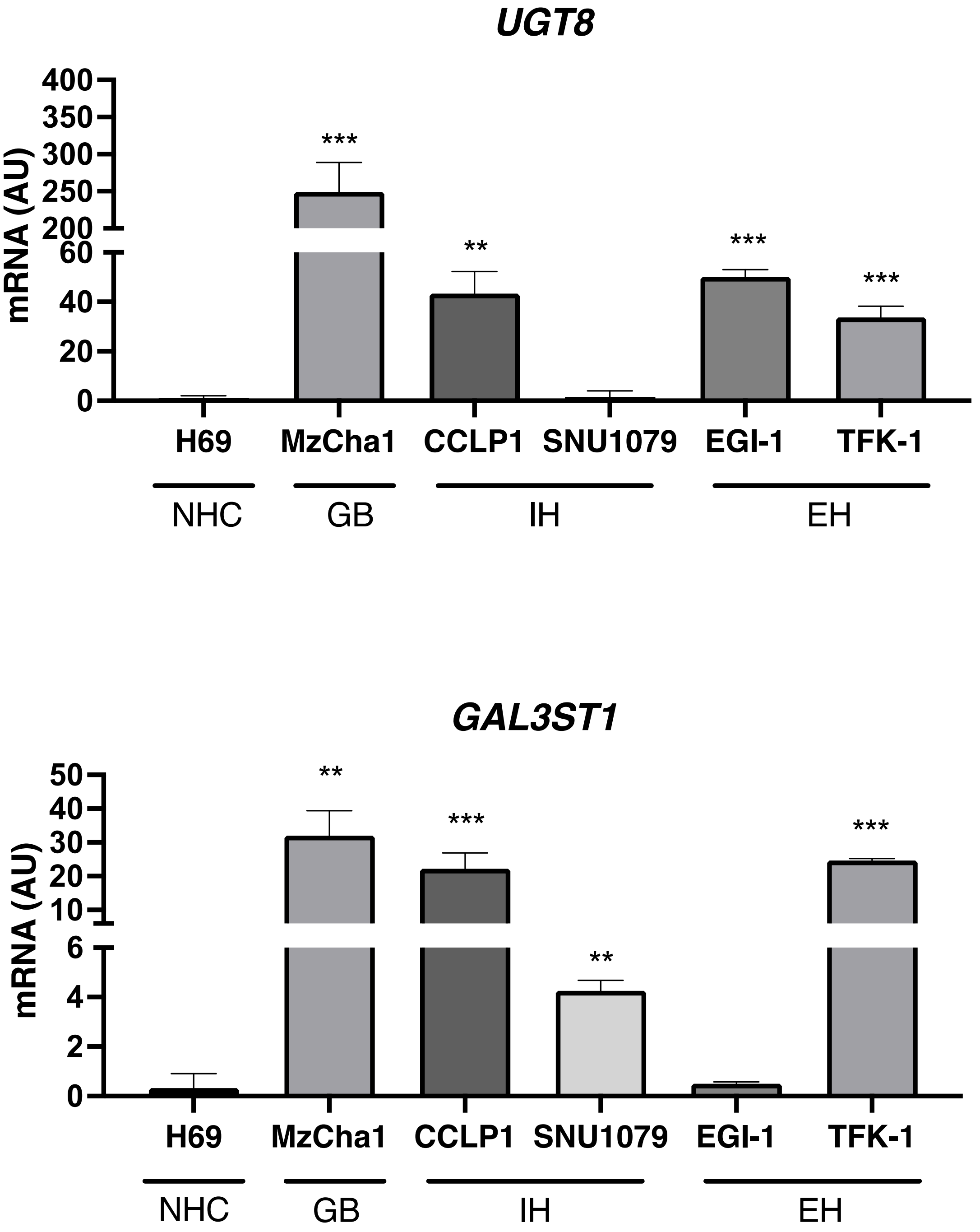

Figure S1B. *UGT8* and *GAL3ST1* expression in human CCA cell lines. Relative mRNA levels of *UGT8* and *GAL3ST1* in human CCA cell lines, including extrahepatic (EH) CCA (TFK1, EGI-1), intrahepatic (IH) CCA (CCLP1, SNU1079), and gallbladder (GB) carcinoma (Mz-Cha-1) cell lines compared to normal human cholangiocyte (NHC) cell line (H69). Data are shown as mean ± SD. **$p < 0.01$, ***$p < 0.001$ vs. H69.

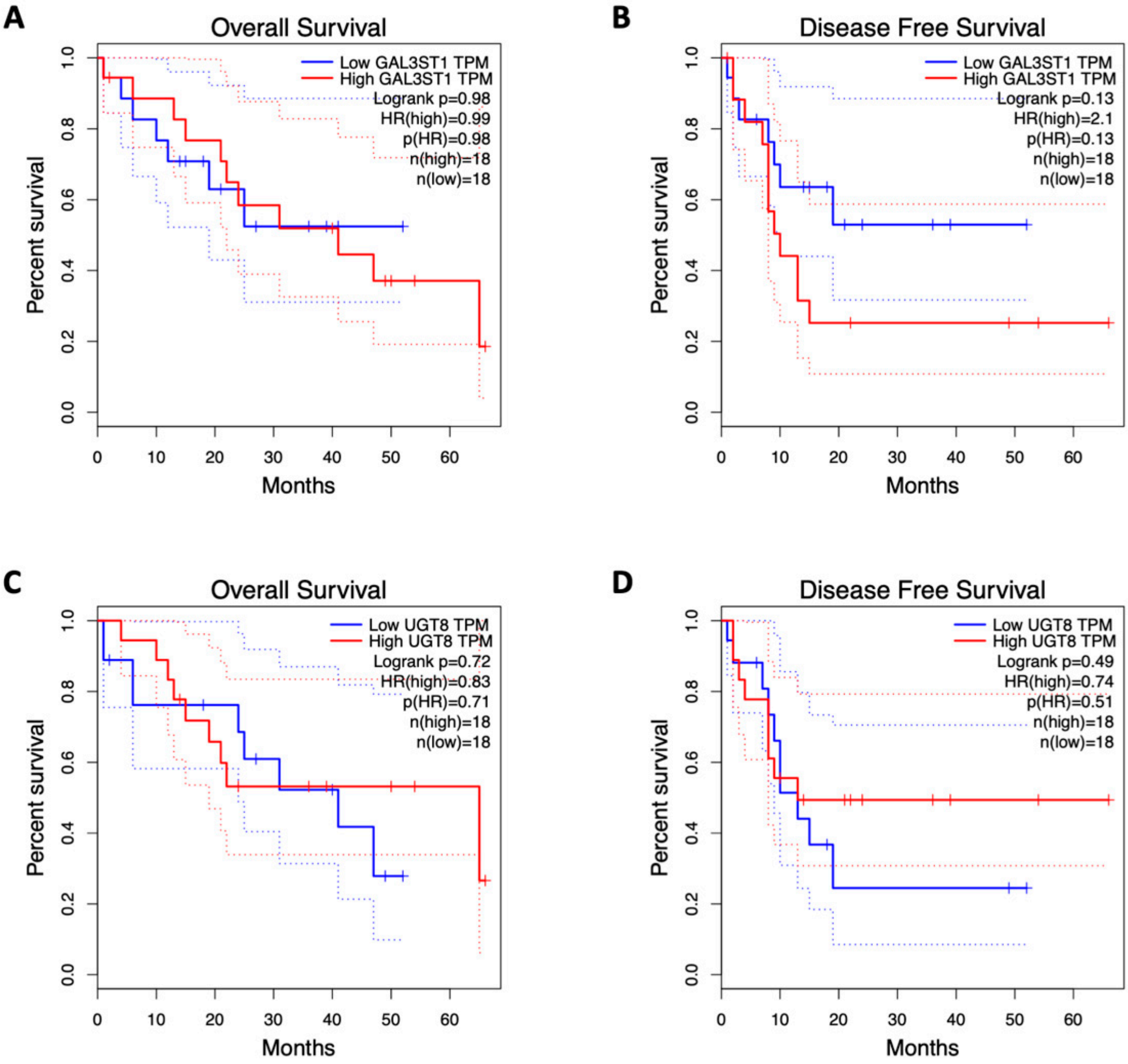

Figure S1C. Overall survival (OS) analysis and disease free survival (RFS) analysis of *GAL3ST1* (A and B) and *UGT8* (C and D) with GEPIA database. Patients were divided into high and low expression groups based on the median expression level of each gene.; Each group contains 18 patients (*n* = 18).

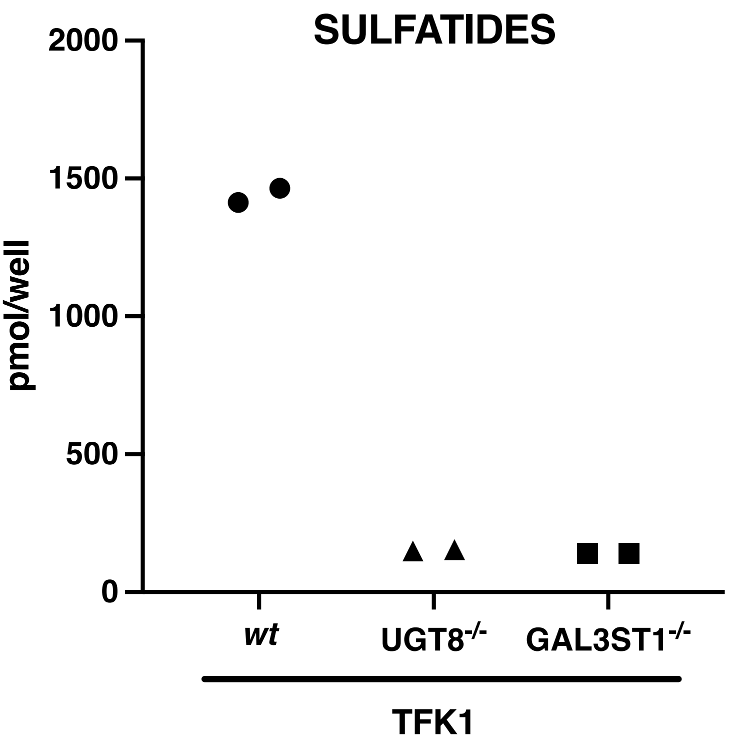

Figure S2. Sulfatide levels in TFK1 *wt, UGT8$^{-/-}$* and *GAL3ST1$^{-/-}$* cells. Total sulfatide levels were determined by LC-MS analysis of cell lysates of TFK1 *wt*, *UGT8$^{-/-}$* and *GAL3ST1$^{-/-}$* cells; *n* = 2.
